# Proteolysis in human breast and colorectal cancer

**DOI:** 10.1038/sj.bjc.6690689

**Published:** 1999-09

**Authors:** E A Garbett, M W R Reed, N J Brown

**Affiliations:** Professional Unit of Surgery, North Tees General Hospital, Stockton-on-Tees, TS19 8PE, UK; Departments of Surgical and Anaesthetic Sciences, Surgery, Central Sheffield University Hospitals, Floor K, Royal Hallamshire Hospital, Sheffield, S10 2JF, UK

**Keywords:** proteolysis, matrix metalloproteinases (MMPs), breast cancer, colorectal cancer, metastasis

## Abstract

Proteolysis occurs when proteinase activity exceeds inhibitor activity. Proteolysis is normally tightly regulated and is involved in cancer invasion and metastasis. The aim of this study was to compare proteolysis in breast and colorectal cancer. Proteinase and inhibitor expression were analysed in paired tumour and normal tissue samples from 43 breast and 24 colorectal cancer patients using substrate zymography, Western blotting and quenched fluorescence substrate hydrolysis. The expression of the latent forms of matrix metalloproteinase-2 (MMP-2), MMP-3 and MMP-9, urokinase plasminogen activator (uPA), tissue inhibitor of metalloproteinase-1 (TIMP-1) and TIMP-2 expression were observed in both tumour and normal tissue samples from breast and colorectal tissue; however, expression was greater in the tumour tissue. Expression of active MMP-2 and MMP-9 and the total MMP activity were greater in tumour compared to normal samples in both tissues (*P*< 0.05). The expression of all proteinases and total MMP activity was greater in colorectal tissue than breast tissue samples. Breast and colorectal cancer demonstrated different proteinase profiles, however proteolysis in both tissues was greater in tumour tissue than normal tissue. © 1999 Cancer Research Campaign


*British Journal of Cancer* (1999) **81**(2), 287–293
© 1999 Cancer Research Campaign
Article no. bjoc.1999.0689

### Proteolysis in human breast and colorectal cancer

**EA Garbett[1], MWR Reed[3] and NJ Brown[2]**

[1]Professional Unit of Surgery, North Tees General Hospital, Stockton-on-Tees TS19 8PE, UK; Departments of [2]Surgical and Anaesthetic Sciences, and [3]Surgery, Central Sheffield University Hospitals, Floor K, Royal Hallamshire Hospital, Sheffield S10 2JF, UK

**Summary** Proteolysis occurs when proteinase activity exceeds inhibitor activity. Proteolysis is normally tightly regulated and is involved in cancer invasion and metastasis. The aim of this study was to compare proteolysis in breast and colorectal cancer. Proteinase and inhibitor expression were analysed in paired tumour and normal tissue samples from 43 breast and 24 colorectal cancer patients using substrate zymography, Western blotting and quenched fluorescence substrate hydrolysis. The expression of the latent forms of matrix metalloproteinase-2 (MMP-2), MMP-3 and MMP-9, urokinase plasminogen activator (uPA), tissue inhibitor of metalloproteinase-1 (TIMP-1) and TIMP-2 expression were observed in both tumour and normal tissue samples from breast and colorectal tissue; however, expression was greater in the tumour tissue. Expression of active MMP-2 and MMP-9 and the total MMP activity were greater in tumour compared to normal samples in both tissues ($P < 0.05$). The expression of all proteinases and total MMP activity was greater in colorectal tissue than breast tissue samples. Breast and colorectal cancer demonstrated different proteinase profiles, however proteolysis in both tissues was greater in tumour tissue than normal tissue.

© 1999 Cancer Research Campaign

**Keywords:** proteolysis; matrix metalloproteinases (MMPs); breast cancer; colorectal cancer; metastasis

The co-ordinated synthesis and degradation of the extracellular matrix (ECM) maintains structural organization of connective tissues. ECM degradation by proteinases is involved in both physiological, e.g. wound healing and bone remodelling, and pathological processes, e.g. cancer and arthritis. The difference in ECM degradation in these processes is that it is normally tightly regulated and the normal matrix boundaries are preserved (Khokha et al, 1995). However, in pathological processes, such as tumour invasion and metastasis, ECM turnover is not regulated normally resulting in loss of matrix function and compromised matrix boundaries (Cocoran et al, 1996). The proteinases involved in tissue degradation are subject to strict regulatory mechanisms including their synthesis, secretion, catalytic activity and the existence of specific natural proteinase inhibitors (Testa and Quigley, 1990). In cancer, proteinases are thought to enhance cancer invasion by catalysing the degradation of ECM components. Such degradation occurs at several stages of the metastatic cascade, including angiogenesis, local invasion and intravasation. Since multiple ECM components exist, a number of different proteinases are likely to be required to complete the metastatic sequence. These proteinases can be subdivided into four distinct sub-classes based on their catalytic mechanism: serine, cysteine, aspartic and matrix metalloproteinases (MMPs) (Barrett, 1995). The proteinases primarily involved in ECM degradation in tumour invasion and metastasis are the MMPs and plasminogen activators.

MMPs or matrixins, are a family of zinc- and calcium-dependent endopeptidases that have the combined ability to break down all ECM components. Fourteen human MMPs exist and these can be grouped according to their domain structure into at least five groups: the collagenases, gelatinases, stromelysins, membrane-type MMPs and others (Baramova and Foidart, 1995). MMPs can exist in latent and active forms and complexed with and without their specific inhibitors, the tissue inhibitors of metalloproteinases (TIMPs). MMP activity is regulated at several levels, including gene expression, secretion, activation and inhibition by the TIMPs. Four human TIMPs have been identified to date (TIMPs 1–4) which have closely related structures and inhibitory properties. TIMPs bind non-covalently to active MMPs forming essentially irreversible complexes inhibiting MMP activity (Cawston et al, 1983). Certain TIMPs also bind to latent forms of MMPs, e.g. TIMP-1 to latent MMP-9 and TIMP-2 to latent MMP-2, thus controlling the activation of these latent MMPs. It is therefore the balance between the levels of activated MMPs and free TIMPs that determines the overall MMP activity and ECM degradation.

Plasminogen activators (PAs) are members of the serine proteinase family and two PAs exist, urokinase-type (uPA) and tissue type (tPA). PAs cleave plasminogen to the active proteinase plasmin, which can catalyse the degradation of a variety of proteins including fibrin and laminin (Kwaan, 1992), as well as activating other proteinases by cleaving the pro-form, e.g. MMP-1 (interstitial collagenase). The catalytic activity of this plasminogen–plasmin system is modulated by PAs and by inhibitors of both plasmin and PAs. Plasmin inhibitors include α-2 antiplasmin and α-2-macroglobulin, and PA inhibitors include the type 1 and 2 plasminogen activator inhibitors (PAIs).

Proteinase expression has been extensively studied in many different human cancers including breast (Schmitt et al, 1991; Clavel et al, 1992; Brown et al, 1993; Davies et al, 1993; Duffy et al, 1993; Remacle et al, 1998), colorectal (Hewitt et al, 1991; Liabakk et al, 1996; Parsons et al, 1998), prostate (Hamdy et al, 1994) and stomach (Torii et al, 1997). These studies used a variety of techniques, including immunohistochemistry, zymography and enzyme-linked immunosorbent assays (ELISAs); however, no previous study has compared the factors involved in proteolysis in different cancers, or used a variety of techniques to do so.

*Received 19 November 1998*
*Revised 24 March 1999*
*Accepted 30 March 1999*

*Correspondence to:* EA Garbett

As tumours are heterogeneous, the stromal components within tumours and the surrounding ECM may vary in different tissues. Therefore different proteinases may be involved for successful invasion and metastasis in different human tumours. The aim of the current study was therefore to compare the process of proteolysis in two human cancers, breast and colorectal cancer. To determine whether there were differences in proteolysis in these cancers, three different techniques were employed and these determined three different aspects of proteolysis. First, substrate zymography determined the expression of different proteinases (MMPs and PAs) in paired tumour and normal tissue samples from patients undergoing surgical resection for breast and colorectal cancer. Secondly, Western blotting compared the expression of inhibitors (TIMP-1 and -2) in the same samples and finally quenched fluorescence substrate hydrolysis determined total ‘free’ MMP activity and therefore potential ECM degradation of each tissue sample.

### MATERIALS AND METHODS

### Tissue samples

Paired fresh tumour and normal breast ($n$ = 43) and colorectal ($n$ = 24) tissue samples were collected in RPMI media by a consultant histopathologist from Histopathology Department, Royal Hallamshire Hospital, Sheffield after surgical resection for breast and colorectal cancer. The tissue samples were mechanically disaggregated using scalpel blades and graded needles, to yield a single cell suspension. The cell suspension was centrifuged at 2100 rpm for 10 min. The cell pellet was resuspended and the viable cells were counted using a haemocytometer (Neubauer, Phillip Harris Scientific). After counting, the cell pellet was reformed and the cells resuspended in lysis buffer (0.1% Triton X-100 in 0.05 M trizma base, 0.2 M sodium chloride (NaCl) and 0.005 M calcium chloride ($CaCl_2$) at a concentration of $10 \times 10^6$ cells per ml buffer.

### Zymography

Before substrate zymography, the tissue sample lysates were mixed 3:1 with non-reducing sample buffer (0.5 M Tris–HCI, pH 6.8, sodium dodecyl sulphate (SDS), glycerol and bromophenol blue).

#### *Gelatin zymography*

Gelatin zymography was performed to determine the expression of gelatinase A (MMP-2) and gelatinase B (MMP-9) in the different tissue samples (Heussen and Dowdle, 1980). Each sample (20 μl) was run in parallel with a molecular weight marker and MMP-2 and MMP-9 protein standards (where available, TCS Biologicals, UK) on SDS-polyacrylamide gels (7.5%) containing 0.1% gelatin as the substrate. Gels were electrophoresed at 200 V for 1 h (mini-V 8.10; BRL Life Technologies, UK). After electrophoresis, the gel was washed in 2% Triton X-100 for 1 h at room temperature on an orbital shaker. The substrate gel was then incubated overnight with MMP incubation buffer (0.05 M trizma base, 0.2 M NaCl, 5 mM $CaCl_2$). Following incubation, gels were stained with 0.2% solution Coomassie blue for 15 min and then destained (10% acetic acid and 30% methanol) for 10 min. Proteolytic activity was represented by clear lysis bands of degraded protein on a uniformly blue background.

#### *Casein zymography*

The presence of stromelysin-1 (MMP-3) was determined using a 12% SDS-polyacrylamide substrate gel containing 0.1% casein (Seftor, 1994).

#### *Collagen I zymography*

The presence of interstitial collagenase (MMP-1) was determined using a substrate gel containing 0.1% collagen type I in 12% SDS-polyacrylamide gel.

#### *Control gels for MMPs*

Control gels contained either of the MMP inhibitors, EDTA or 1,10 phenanthroline in the MMP incubation buffer to confirm that the lysis bands were due to MMPs.

#### *Double substrate zymography*

Double substrate zymography was used to determine the presence of PAs in tissue samples (Seftor, 1994). The two substrates used were plasminogen (substrate 1) and gelatin (substrate 2). The plasminogen acts as a substrate for any PAs present in the sample, by cleaving plasminogen to the active enzyme, plasmin, which subsequently degrades the gelatin. The PA incubation buffer (0.25 M trizma base, pH 8.1) contained EDTA to eliminate any gelatinase activity in the sample on the substrate gelatin.

#### *Control gels for PAs*

Each sample was also run down two control gels, the first gel only contained gelatin as a substrate, therefore any PAs present in the samples would be unable to degrade the gelatin, and the second gel contained the serine proteinase inhibitor phenylmethylsulphonyl fluoride in the incubation buffer to determine if lysis bands were due to PAs.

#### *Quantitation of the gels*

Gels were quantitated using laser densitometry. Gels were scanned and analysed using the Quantity One software (Discovery Series, Pharmacia Biotech, UK). The image of the gel was inverted to reveal dark bands on a white background. The molecular weight, area and optical density of each band were determined. The relative proteinase activity was determined for each proteinase by multiplying the area of each band by its optical density.

### Western blotting

Western blotting was performed on the same breast and colorectal tissue samples to determine the expression of the TIMPs, TIMP-1 and TIMP-2. Samples were run down SDS polyacrylamide gels (15%) and electrophoresed at 200 V for 1 h. Control TIMP proteins (TIMP-1 and TIMP-2; Calbiochem, UK) were run in parallel with tissue samples. Proteins within the gel were then blotted onto a nitrocellulose membrane following electrophoresis at 150 V for 1 h. Once protein transfer was completed the membrane was incubated with blocking agent for 1 h to stop any non-specific binding. After washing, the membrane was incubated with the primary antibody (TIMP-1 or TIMP-2 mouse anti-human antibodies; 1 μg $ml^{-1}$ TIMP-1 or 5 μg $ml^{-1}$ TIMP-2; Calbiochem UK), for 1 h. It was then rinsed and incubated with the secondary antibody (anti-mouse peroxidase labelled secondary antibody) for 1 h.

#### *Detection and analysis*

The membranes were washed and drained and the detection reagent added to the protein side of the membrane for 1 min.

© 1999 Cancer Research Campaign

Excess reagent was removed and the membrane was placed protein side down onto a piece of Sarawrap (plastic tool wrap). The membrane was then exposed to an autoradiography film and the film was developed. The protein bands on the processed film were analysed by densitometry.

### Quenched fluorescence substrate hydrolysis

The technique of quenched fluorescence substrate hydrolysis employs the quenched fluorescence substrate Mca-Pro-Leu-Gly-Leu-Dpa-Ala-Arg-$NH_2$ which is cleaved by all secreted activated MMPs, so far tested (Brown et al, 1996), at the Gly-Leu bond releasing the fluorescent Mca group from the internal quenching group Dpa (Knight et al, 1992). The total MMP activity was determined in all tissue samples by incubating 150-µl tissue sample lysate with 2835-µl assay buffer (0.1 M Tris–HCl, 0.1 M NaCl, 10 mM $CaCl_2$, pH 7.5) and 15 µl of the fluorescent substrate (5 µM). The samples were incubated for 3 h at 37°C and the MMP activity was determined on a fluorimeter (Perkin-Elmer LS50B) ($\lambda_{ex}$ 328 nm and $\lambda_{em}$ 393 nm) running the FLDM software. A total of 150 µl lysis buffer incubated as above acted as the negative control.

The fluorimeter was standardized (maximum fluorescence was set by the addition of 0.5-µM standard, Mca-Pro-Leu-OH, the fluorescent product produced) so that a comparative rate of substrate hydrolysis was determined for each sample and expressed as pM $min^{-1}$.

### Statistical analysis

For comparisons between proteinase and inhibitor expression in breast and colorectal, tumour and normal tissue samples, the Mann–Whitney *U*-test for non-parametric data, with 95% confidence limits was performed. Differences were considered to be significant at $P < 0.05$ level.

### RESULTS

There was a wide variation in the proportion of breast and colorectal samples expressing each proteinase and inhibitor (Table 1) as well as the amount of each protein expressed (Table 2).

### Proteinase expression by substrate zymography

Colorectal and breast tissues, as well as tumour and normal tissue samples exhibited differential proteinase profiles. After zymography, the number of samples expressing each MMP lysis band were determined by running the relevant MMP control protein in parallel down the substrate zymogram. In both tissue types, the greatest difference in proteinase expression was observed after gelatin zymography.

#### *MMP-2 and MMP-9 expression*

Gelatin zymography identifies and separates the gelatinases MMP-2 and MMP-9 in both latent and active forms due to differences in their molecular mass. In both breast and colorectal tissue the following four lysis bands were observed in the samples: 92 kDa corresponding to latent MMP-9; 84 kDa active MMP-9; 72 kDa latent MMP-2, and finally 68 kDa active MMP-2.

Latent MMP-2 and latent MMP-9 expression was observed in a similar proportion of tumour and normal tissue samples in both breast and colorectal tissues – latent MMP-2: 100% breast tumour samples, 100% normal breast, 100% colorectal tumours and 92% normal colorectal samples; latent MMP-9: 100% breast tumours, 93% normal breast, 100% colorectal tumours and 100% normal colon samples (Table 1). However, the amounts of these enzymes expressed were significantly greater in tumour tissue than the corresponding normal tissue samples (Table 2; $P < 0.05$). The major difference in MMP expression between tumour and normal tissue was in the expression of active MMP-2 and active MMP-9 (Tables 1 and 2). Both the proportions of tissue samples expressing these enzymes and the amounts of these active enzymes expressed, were significantly greater in the tumour tissue in both breast and colorectal samples ($P < 0.05$). Active MMP-2 was expressed by 98% breast tumours, 49% normal breast samples, 100% colorectal tumours and 50% normal colon samples; active MMP-9 was expressed by 78% breast tumours, 7% normal breast samples, 100% colorectal tumours and 13% normal colon samples. Figure 1 demonstrates representative gelatin zymograms for breast and colorectal tissue samples.

#### *MMP-3 expression*

Following casein zymography, two different lysis bands were observed, latent MMP-3 migrating at a molecular mass of 57 /59 kDa and active MMP-3 at 45 kDa. Both latent and active MMP-3 were expressed in a significantly greater number of colorectal and breast tumours when compared with the corresponding normal tissue samples ($P < 0.05$, Table 1). Latent MMP-3 was expressed in a greater proportion of breast tumours than colorectal tumours (73% breast tumours compared to 56% colorectal tissues) and active MMP-3 was expressed in a greater proportion of colorectal tissue samples than breast (92% colorectal tumour samples compared to 41% breast tumour samples; Table 1). Latent MMP-3 was more widely expressed in breast tissue than active MMP-3; the opposite was seen with colorectal tissue, where active MMP-3 was expressed in a greater proportion of tumour and normal tissue samples than latent MMP-3 (Table 1).

#### *MMP-1 expression*

Of all the MMPs studied, MMP-1 was expressed the least by any tissue and mainly only in the latent form. MMP-1 was expressed in a greater proportion of tumour than normal tissue samples in both tissues and was also significantly greater in colorectal tissue than breast ($P < 0.05$). MMP-1 was only expressed in 22% breast

**Table 1** Expression of proteinases and inhibitors in breast and colorectal samples: results are expressed as the percentage of samples expressing each proteinase/inhibitor

| Proteinase/ inhibitor | Breast tumour (%) | Normal breast (%) | Colorectal tumour (%) | Normal colon (%) |
|---|---|---|---|---|
| Latent MMP-9 | 100 | 93 | 100 | 100 |
| Active MMP-9 | 78 | 7 | 100 | 13 |
| Latent MMP-2 | 100 | 100 | 100 | 92 |
| Active MMP-2 | 98 | 49 | 100 | 50 |
| Latent MMP-3 | 73 | 16 | 56 | 33 |
| Active MMP-3 | 41 | 19 | 92 | 75 |
| Latent MMP-1 | 22 | 0 | 74 | 32 |
| Active MMP-1 | 12 | 0 | 9 | 5 |
| uPA | 90 | 56 | 100 | 77 |
| TIMP-1 | 82 | 50 | 95 | 77 |
| TIMP-2 | 100 | 80 | 100 | 80 |

© 1999 Cancer Research Campaign

**Table 2** Differences in proteinase and inhibitor expression and MMP activity in breast and colorectal tissue

| | Breast tumour *n* = 43 | Normal breast *n* = 43 | Colorectal tumour *n* = 24 | Normal colon *n* = 24 |
|---|---|---|---|---|
| Latent MMP-9 | 0–33.63 (10.52) | 0–24.91 (6.63) | 3.04–67.56 (26.9)[a] | 0.74–52.48 (20.6)[a] |
| Active MMP-9 | 0–23.46 (11.56) | 0–7.57 (0) | 0.23–59.43 (18.8)[a] | 0–17.06 (0) |
| Total MMP-9 | 0.088–70.62 (11.98) | 0–24.68 (7.14) | 3.27–126.79 (56.20) | 0.74–58.87 (21.98) |
| Latent MMP-2 | 0.27–20.46 (5.74) | 0.23–18.85 (3.22) | 0.34–33.07 (7.3) | 0–13.92 (4.85) |
| Active MMP-2 | 0–34.15 (5.29) | 0–11.78 (0) | 0.03–29.9 (8.3) | 0–7.07 (0.2) |
| Total MMP-2 | 0.27–52.02 (8.3) | 0.23–12.06 (3.57) | 2.02–62.98 (15.04) | 0–17.05 (5.61) |
| Latent MMP-3 | 0–9.2 (5.0) | 0–3.7 (0) | 0–52.08 (0.96) | 0–23.58 (0) |
| Active MMP-3 | 0–27.7 (0) | 0–17.6 (0) | 0–69.08 (15.63)[a] | 0–69.74 (13.48)[a] |
| Total MMP-3 | 0–31.4 (4.00) | 0–17.6 (0) | 0–121.16 (16.71)[a] | 0–82.11 (13.96)[a] |
| Latent MMP-1 | 0–2.55 (0) | 0 (0) | 0–11.04 (2.5)[a] | 0–7.15 (0) |
| Active MMP-1 | 0–3.31 (0) | 0 (0) | 0–8.2 (0) | 0–5.5 (0) |
| Total MMP-1 | 0–5.86 (0) | 0 (0) | 0–17 (5.5)[a] | 0–16.5 (0) |
| uPA | 0–56.00 (6.85) | 0–16 (1.45) | 0.86–21.49 (6.91) | 0–18.34 (3.55)[a] |
| TIMP-1 | 0–224.2 (6.00) | 0–24.3 (1.6) | 4.6–87.2 (15.3)[a] | 0–77.7 (4.15) |
| TIMP-2 | 3.1–36.5 (11.7) | 0–26.4 (7.7) | 4–15.4 (4.9) | 0–3.1 (1.5) |
| Rate of substrate hydrolysis | 0–8325 (808.4) | 0–2245 (225.1) | 791–22470 (2207.5)[a] | 111.4–11 132.8 (978.45)[a] |

Data are presented as the range and median values (in parentheses) for the amounts of each proteinase and inhibitor (in arbitrary units) and the rate of substrate hydrolysis (pM $min^{-1}$). [a]$P < 0.05$ Mann–Whitney *U*-test, colorectal versus breast tumour and normal colon versus normal breast.

tumours and no normal breast tissue samples compared to 74% colorectal tumours and 32% normal colon samples.

### *Plasminogen activator expression*

Double substrate zymography determined PA activity within the different tissue samples. Two PAs exist and they can be separated electrophoretically according to their molecular mass, tPA migrates at 70 kDa and uPA at 54 kDa. The only PA to be expressed in any sample studied migrated at 54 kDa corresponding to uPA. No lysis band was observed migrating at 70 kDa in any tissue. uPA was expressed in a significantly greater proportion of tumours than normal tissue samples in both breast and colorectal cancer ($P < 0.05$); 90% breast tumours, 56% normal breast samples, 100% colorectal tumour samples and 77% normal colon samples (Table 1). uPA expression was greater in both colorectal tissues than the equivalent breast tissue (Table 2), however the difference was only significant when comparing normal tissues ($P < 0.05$).

### TIMP expression by Western blotting

Following Western blotting, TIMP-1 was expressed in a significantly greater proportion of tumour tissue samples than normal tissue in both breast and colorectal tissue ($P < 0.05$). TIMP-1 was expressed by 82% breast tumour samples, 50% normal breast samples, 95% colorectal tumour samples and 77% normal colon samples studied. The amount of TIMP-1 expressed by the tumour tissue was significantly greater in colorectal tissue than the breast ($P < 0.05$) and in normal tissue, TIMP-1 expression was also greater in colorectal tissue; however, the difference was not significant.

The proportion of tissue samples expressing TIMP-2 were the same for both breast and colorectal tissue samples. Although TIMP-2 expression in colorectal tissue was greater than that in breast, the difference was not significant.

### Total MMP activity

The rate of substrate hydrolysis (RSH) following quenched fluorescence substrate hydrolysis is proportional to the total amount of free active MMPs present within the tissue samples. There was great variation in the rates of substrate hydrolysis between both colorectal and breast samples (Figure 2). All colorectal tissue samples successfully cleaved the substrate (i.e. RSH were greater than 0 pM $min^{-1}$), but not all breast samples exhibited substrate hydrolysis. However, in both tissues the RSH was significantly greater in the tumour tissue than the corresponding normal ($P < 0.05$).

 *© 1999 Cancer Research Campaign*

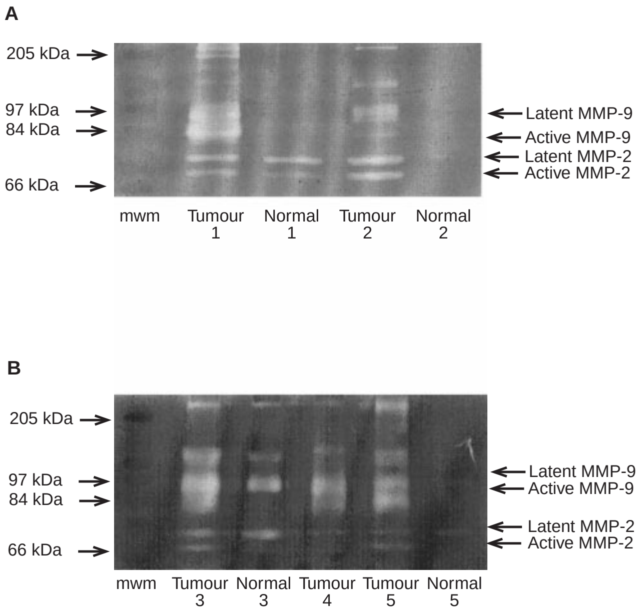

**Figure 1** Gelatin zymograms illustrating the gelatinolytic activity of (**A**) two paired breast tumour and normal tissue samples and (**B**) three colorectal tumour samples and two normal colon samples. Lane 1 corresponds to the molecular weight marker (mwm), the remaining lanes correspond to the different tissue samples. All tumour samples (breast and colon) expressed both the latent and active forms of MMP-2 and MMP-9. One normal breast sample (Normal 1) expressed latent and active MMP-2 but no MMP-9. The other normal samples (breast and colon, samples 2, 3 and 5) expressed fainter bands corresponding to latent MMP-2 and MMP-9

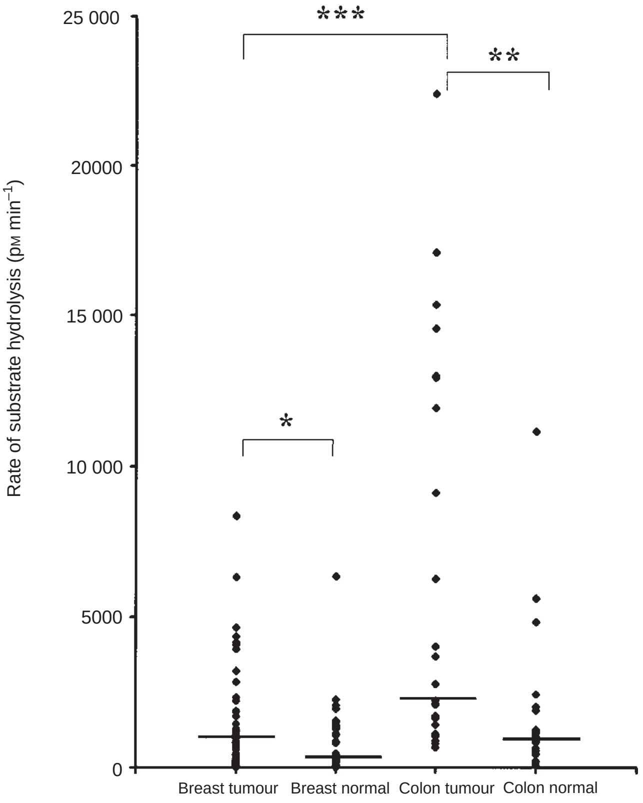

**Figure 2** Graph demonstrating the differences in the rate of substrate hydrolysis (MMP activity) in breast and colorectal tumour and normal tissue samples. The line corresponds to the median value for each tissue type. The rate of substrate hydrolysis was significantly greater in tumour than normal tissue samples in both breast (*$P < 0.05$, Mann–Whitney *U*-test) and colorectal (**$P < 0.05$) tissue. The rate of substrate hydrolysis was significantly greater in colorectal tissue than breast (***$P < 0.05$)

The RSH was significantly greater in colorectal tissue than breast tissue ($P < 0.05$) and this can be clearly observed with the range and median values for the RSH (Table 2). For breast tumour tissue the RSH ranged from 0 where samples exhibited no MMP activity to 8325 pM min$^{-1}$ and normal breast from 0 to 2245 pM min$^{-1}$. The median values were 808 and 225 pM min$^{-1}$ respectively. However, for colorectal tumour tissue, the range was from 791 to 22 470 pM min$^{-1}$ and for normal colorectal tissue from 111 to 11 133 pM min$^{-1}$ with median values of 2207 and 978 pM min$^{-1}$ respectively.

### DISCUSSION

This study has examined proteolysis, one of the important processes involved in tumour cell invasion and metastasis. Under normal conditions, proteolytic enzymes are tightly controlled by specific proteinase inhibitors; the MMPs are regulated by TIMPs and PAs are regulated by PAIs. It is thought to be the balance between these proteinases and inhibitors that determines the occurrence of proteolysis in vivo. If proteinase expression increases and/or the inhibitor expression decreases then the balance probably favours proteolysis.

No single study described to date has compared proteolysis in different cancers and few have studied proteinase and inhibitor expression using more than one technique. In the present study, proteolysis was compared in breast and colorectal cancer employing three complementary techniques: zymography to determine proteinase expression, Western blotting to identify TIMPs and quenched fluorescence substrate hydrolysis to determine the total MMP activity within the samples.

Previously, studies have homogenized the tissue samples (Ganesh et al, 1997; Parsons et al, 1998); however, in the present study, the tissue was mechanically disaggregated, cells counted and then lysed. The major advantage of this method is that the same number of cells were present in each sample analysed rather than using an equivalent weight of tissue as described in previous studies. Therefore any differences observed in proteinase and inhibitor expression between samples will be due to differences in the relative proportions of cells, e.g. tumour cells compared to stromal cells, and/or the tissue's ability to produce, secrete or activate these factors. Differences will not be due to cell numbers. However, the disadvantage of using cell lysates compared to solid tissue homogenates to determine proteinase expression, is that the results are likely to be biased towards secreted proteinases, e.g. MMP-9 and those that have cell surface receptors, e.g. MMP-2 or uPA. It is unlikely that proteinases bound to the ECM, e.g. tPA, will be identified by this method.

The present study described the presence of multiple proteinases in normal and malignant breast and colorectal tissue. Furthermore, differential proteinase profiles were observed in normal and tumour tissue in both breast and colorectal samples. The expression of all proteinases identified (MMP-1, -2, -3, -9 and uPA) was greater in tumours than normal tissue, in both breast and colorectal samples. The expression of these proteinases also tended to be greater in colorectal tissue than breast. The most marked difference in proteinase expression was observed following gelatin zymography where the expression of the active forms of MMP-2 and MMP-9 were significantly greater in tumours when compared to normal breast or colon. The number of colorectal and breast samples expressing MMP-2 and -9 in the latent and active forms in both tumour and normal tissue samples were similar for both tissues. However, the amount expressed (latent, active and total) was greater in colorectal tissue than in breast. Previously, two independent studies using zymography determined comparable mean values for MMP-2 and MMP-9 expression in colorectal and breast cancer tissue, with MMP-9 expression being slightly higher in the colon (Liabakk et al, 1996). Previous studies employing gelatin zymography on breast (Brown et al, 1993; Davies et al, 1993; Remacle et al, 1998) and colorectal (Liabakk et al, 1996; Parsons et al, 1998) cancer have demonstrated similar results for MMP-2 expression to the present study. However, in contrast to these studies, the present study identified active MMP-9 expression in a greater proportion of tumour samples (breast and colorectal). Possible explanations for these discrepancies are the disaggregation technique employed, the amount of tissue collected, the grade of tumour and the area of the tumour from which the tissue sample was taken.

Few studies have determined MMP-1 or MMP-3 expression in either breast (Polette et al, 1993; Heppner et al, 1996; Remacle et al, 1998) or colorectal cancer (Matrisian et al, 1994; Gallegos et al, 1995; Murray et al, 1996), and no study has compared the expression of these MMPs in both tissues. Latent MMP-3 alone was expressed by a greater proportion of breast samples than colorectal. However, the total amount of MMP-3 expressed (latent + active) was greater in colorectal tissue. Active MMP-3 was expressed by a greater proportion of colorectal tumour and normal tissue samples than breast tissue samples. MMP-1 was the least expressed MMP. MMP-1 expression was differential, with a greater proportion of tumours expressing MMP-1 when compared to normal tissue samples, and greater expression in colorectal versus breast. Few studies investigating proteinase profiles in cancer have identified MMP-1 expression. In breast cancer, the techniques previously employed were immunohistochemistry (Clavel et al, 1992), ELISA (Remacle et al, 1998) and Northern blotting (Polette et al, 1993) and in agreement with the current study, MMP-1 expression was low and not consistently observed. In colorectal cancer, two studies have employed immunohistochemistry to determine MMP-1 expression, one study has shown an association between MMP-1 expression and poor prognosis (Murray et al, 1996); however, in contrast another study observed no MMP-1 expression in colorectal tumours (Gallegos et al, 1991).

The only PA detected in any tissue was uPA; however, the absence of tPA may reflect the tissue disaggregation technique employed in this study. The increased uPA expression in tumour tissue is consistent with previous studies for both breast (Janicke et al, 1992, 1993; Bouchet et al, 1994) and colorectal cancer (Grondahl-Hansen et al, 1991; Pyke et al, 1991; Buo et al, 1995). However, no previous study has compared PA expression in breast and colorectal tissues. uPA expression in these studies was confined to the stromal cells; however, the receptor for uPA, uPAR, was expressed by tumour cells. tPA expression was also observed in a few endothelial cells in both tumour and normal tissue in one study (Grondahl-Hansen et al, 1991). There was also variation in TIMP expression in breast and colorectal tissues as determined by Western blotting. In both breast and colorectal samples, TIMP-1 expression was greater in tumour than normal tissue, but TIMP-1 expression was around threefold greater in both colorectal tumour and normal tissue when compared to the equivalent breast tissue. In contrast, TIMP-2 expression in tumour and normal tissues was greater in breast than colorectal tissue, but greater in tumour compared to normal for both tissues.

Both proteinase and inhibitor expression were found to be up-regulated in tumour tissue in breast and colorectal cancer; however, the most important determinant for proteolysis in vivo is the balance between the expression and activation of proteinases and the expression of their inhibitors. Quenched fluorescence substrate hydrolysis determined the amount of free active MMPs present within the tissue samples. This is indicative of proteolysis occurring in vivo and has not been previously determined in either breast or colorectal cancer. In both cancers the total MMP activity was significantly greater in the tumour tissue and this may be relevant in tumour invasion and metastasis. MMP activity was greater in colorectal tumour and normal tissue than breast samples. The active forms of MMP-2, -3 and -9 were all expressed in greater amounts in colorectal tissue and will therefore contribute to the increased activity. Normal colorectal tissue had a greater degree of MMP activity (RSH) than either the breast tumour or normal tissue. This suggests that the colon has a greater inherent rate of tissue turnover than breast tissue (tumour and normal), or that MMPs within the colon are involved in other physiological processes, not just tissue remodelling. Another possible explanation is that the colon may contain other MMPs not present in the breast and not determined in this study.

In both breast and colorectal tumours, there was a wide variation in the amounts of each proteinase and inhibitor expressed. A possible explanation for this may be the varying amounts of stromal components in the different tumours as some proteinases are secreted by stromal cells, e.g. MMP-2 by fibroblasts. Therefore the different proportions of each cell type present within each tumour requires consideration. If the proteinases are only secreted when active proteolysis occurs, then not all tumours will necessarily have an increased proteinase expression at the time of resection. Individual tumours within the same tissue type may rely on different proteinases to degrade the ECM depending on the stage of progression, which may explain the wide variation observed in proteinase expression. Another possible explanation is the differences in the pathological stage/grade of each tumour. The grades of tumour were known for 36/43 breast tumours studied; however, due to the small colorectal sample numbers ($n = 24$) no attempt was made to correlate expression with the pathological stage in the present study. The expression of MMP-2 and MMP-9 appeared to correlate with the grade of breast tumour – grade 1 tumours had a lower expression than grade 3, but an inverse correlation was observed with breast tumour grade and MMP activity; the majority (20/36) of breast tumours were grade 2 compared to grade 1 (6/36) and grade 3 tumours (10/36) (data not shown). The sample sizes for each grade are not equivalent, making statistical comparisons inappropriate at this stage. Therefore a greater number of both breast and colorectal samples need to be analysed before firm conclusions can be determined between proteinase expression and tumour grade.

Although this study identified and quantified a number of proteolytic enzymes that are present in the tumour environment and potentially involved in tumour progression including MMPs and serine proteinases, PAs, other enzymes have also been reported to be involved in this process including MMP-7 and MMP-11 (Liabakk et al, 1996). As it is, the balance between activated proteinases and their inhibitors that modulates tumour invasion, measurements of these other enzymes may provide further

 *© 1999 Cancer Research Campaign*

evidence for the involvement of proteolysis in tumour invasion and metastasis. The tumour expression of these other MMPs are likely to cleave the fluorescent substrate and this may explain the discrepancies observed in proteinase expression in the present study.

In summary, the results presented here demonstrate increased expression of some proteinases in tumour tissue when compared to normal tissue from breast and colorectal cancers. This proteinase expression as well as total MMP activity was greater in colorectal tissue than breast, implying that individual proteinases have differential roles in both physiological and pathological processes in different tissues.

The increased proteolysis observed in both colorectal and breast tumour tissue may be important in invasion and metastasis, since proteinases are involved at several stages of the metastatic cascade including angiogenesis, local invasion, intravasation and extravasation. For example, proteinase inhibitors have been used to try and inhibit angiogenesis in an attempt to prevent and slow down tumour progression (Taraboletti et al, 1995; Conway et al, 1996; Stonelake et al, 1997; Yu et al, 1997). A better understanding of the proteinases and inhibitors involved in tumour progression may allow for therapeutic intervention at the earlier stages of tumour progression.

### REFERENCES

Baramova E and Foidart JM (1995) Matrix metalloproteinase family. *Cell Biol Int* **19**: 239–242

Barrett AJ (1995) Classification of peptidases. *Methods Enzymol* **244**: 1–15

Bouchet C, Spyratos F, Martin PM, Hacene K, Gentile A and Oglobine J (1994) Prognostic value of urokinase type plasminogen activator (uPA) and plasminogen activator inhibitors PAI-1 and PAI-2 in breast carcinomas. *Br J Cancer* **69**: 398–405

Brown CJ, Rahman S, Morton AC, Beauchamp CL, Bramwell H and Buttle DJ (1996) Inhibitors of collagenase but not gelatinase reduce cartilage explant proteolglycan breakdown despite only low levels of matrix metalloproteinase activity. *J Clin Pathol: Mol Pathol* **49**: M331–M339

Brown PD, Bloxidge RE, Anderson E and Howell A (1993) Expression of activated gelatinase in human invasive breast carcinoma. *Clin Exp Metastasis* **11**: 183–189

Buo L, Meling GI, Karlsrud TS, Johansen HT and Aasen AO (1995) Antigen levels of urokinase plasminogen activator and its receptor at the tumour–host interface of colorectal adenocarcinomas are related to aggressiveness. *Human Pathol* **26**: 1133–1138

Cawston TE, Murphy GM, Mercer E, Galloway WA, Hazleman BL and Reynolds JJ (1983) The interaction of purified rabbit bone collagenase with purified rabbit bone metalloproteinase inhibitor. *Biochem J* **211**: 313–318

Clavel C, Polette M, Doco M, Binninger I and Birembaut P (1992) Immunolocalisation of matrix metalloproteinases and their tissue inhibitor in human mammary pathology. *Bull Cancer* **79**: 261–270

Cocoran ML, Hewitt RE, Kleiner DE and Stetler-Stevenson WG (1996) MMP-2: expression, activation and inhibition. *Enzyme Protein* **49**: 7–19

Conway JG, Trexler SJ, Wakefield JA, Marron BE, Emerson DL, Bickett DM, Deaton DN, Garrison D, Elder M, McElory A, Wilmott N, Dockerty AJP and McGeehan GM (1996) Effect of matrix metalloproteinase inhibitors on tumour growth and spontaneous metastasis. *Clin Exp Metastasis* **14**: 115–124

Davies B, Miles DW, Happerfield LC, Naylor MS, Bobrow LC, Rubens RD and Balkwill FR (1993) Activity of type IV collagenases in benign and malignant breast tissue. *Br J Cancer* **76**: 1126–1131

Duffy MJ, Reilly D, O' Sullivan C, O'Higgins N and Fennelly JJ (1990) Urokinase plasminogen activator and prognosis in breast cancer. *Lancet* **335**: 108–111

Gallegos NC, Smales C, Savage FJ, Hembry RM and Boulos PB (1995) The distribution of matrix metalloproteinases and tissue inhibitor of metalloproteinases in colorectal cancer. *Surg Oncol* **4**: 111–119

Grondahl-Hansen J, Ralfkiaer E, Kirkeby, Kristensen P, Lund LT and Dano K (1991) Localisation of urokinase type plasminogen activator in stromal cells in adenocarcinomas of the colon in humans. *Am J Pathol* **138**(1): 111–117

Hamdy FC, Fadlon EJ, Cottam D, Lawry J, Thurrell W, Silcocks PB, Anderson JB, Williams JL and Rees RC (1994) Matrix metalloproteinase 9 expression in primary human prostatic adenocarcinoma and benign prostatic hyperplasia. *Br J Cancer* **69**: 177–182

Heppner KJ, Matrisian LM, Jensen RA and Rodgers WH (1996) Expression of most matrix metalloproteinase family members in breast cancer represents a tumour induced host response. *Am J Pathol* **149**: 273–282

Heussen C and Dowdle EB (1980) Electrophoretic analysis of plasminogen activators in polyacrylamide gels containing sodium dodecyl sulfate and copolymerised substrates. *Anal Biochem* **102**: 196–202

Hewitt RE, Leach IH, Powe DG, Clark IM, Cawston TE and Turner DR (1991) Distribution of collagenase and tissue inhibitor of metalloproteinases (TIMP) in colorectal tumours. *Int J Cancer* **49**: 666–672

Janicke F, Schmitt M and Graeff H (1992) Clinical relevance of the urokinase-type plasminogen activators and their type I inhibitor in breast cancer. *Semin Thromb Hemost* **17**: 303–312

Janicke F, Schmitt M, Pache L, Ulm K, Harbeck N, Hofler H and Graeff H (1993) Urokinase (uPA) and its inhibitor PAI-1 are strong prognostic factors in node negative breast cancer. *Breast Cancer Res Treat* **24**: 195–208

Khokha R, Martin DC and Fata JE (1995) Utilization of transgenic mice in the study of matrix degrading proteinases and their inhibitors. *Cancer Metastasis Rev* **14**: 97–111

Knight CG, Willenbrock F and Murphy G (1992) A novel coumarin-labelled peptide for sensitive continuous assays of the matrix metalloproteinases. *FEBS* **296**: 263–266

Kwaan HC (1992) The plasminogen–plasmin system in malignancy. *Cancer Metastasis Rev* **11**: 291–311

Liabakk NB, Talbot I, Smith RA, Wilkinson K and Balkwill F (1996) Matrix metalloproteinase 2 (MMP-2) and matrix metalloproteinase 9 (MMP-9) type IV collagenases in colorectal cancer. *Cancer Res* **56**: 190–106

Matrisian LM, Wright J, Newell K and Witty JP (1994) Matrix degrading metalloproteinases in tumour progression. *Princess Takamatsu Symposia* **24**: 152–161

Murray GI, Duncan ME, O'Neil P, Melvin WT and Fothergill JE (1996) MMP-1 is associated with poor prognosis in colorectal cancer. *Nat Med* **2**: 461–462

Parsons SL, Watson SA, Collins HM, Griffin NR, Clarke PA and Steele RJC (1998) Gelatinase (MMP-2 and -9) expression in gastrointestinal malignancy. *Br J Cancer* **78**: 1495–1502

Polette M, Clavel C, Cockett M, Girod-de-Bentzmann S, Murphy G and Birembaut P (1993) Detection and localisation of mRNAs encoding matrix metalloproteinases and their tissue inhibitors in human breast pathology. *Invasion Metastasis* **13**: 31–37

Pyke C, Kristensen P, Ralfiaer E, Grondahl-Hansen J, Eriksen J and Dano K (1991) Urokinase-type plasminogen activator is expressed in stromal cells at invasive foci in human colon adenocarcinomas. *Am J Pathol* **138**: 1059–1067

Remacle AG, Noel A, Duggan C, McDermott E, O'Higgins N, Foidart JM and Duffy MJ (1998) Assay of matrix metalloproteinases types 1, 2, 3 and 9 in breast cancer. *Br J Cancer* **77**: 926–931

Schmitt M, Goretzki L, Janicke F, Calvette J, Eulitz M, Kbayashi H, Chucholowski N and Graeff H (1991) Biological and clinical relevance of the urokinase-type plasminogen activator (uPA) in breast cancer. *Biomed Biochim Acta* **50**: 731–741

Seftor REB (1994) Electrophoretic analysis of proteins associated with tumour cell invasion. *Electrophoresis* **15**: 454–462

Stonelake PS, Jones CE, Neoptolemos JP and Baker PR (1997) Proteinase inhibitors reduce basement membrane degradation by human breast cancer cell lines. *Br J Cancer* **75**: 951–959

Taraboletti G, Garofalo A, Belotti D, Drudis T, Borsotti P, Scanziani E, Brown PD and Giavazzi R (1995) Inhibition of angiogenesis and murine hemangioma growth by batimastat, a synthetic inhibitor of matrix metalloproteinases. *J Natl Cancer Inst* **87**: 293–298

Testa JE and Quigley JP (1990) The role of urokinase type plasminogen activator in aggressive tumour cell behaviour. *Cancer Metastasis Rev* **9**: 353–367

Torii A, Kodera Y, Uesaka K, Hirai T, Yasui K, Morimoto T, Yamamura Y, Kato T, Hayakawa T, Fujimoto N and Kito T (1997) Plasma concentration of matrix metalloproteinase 9 in gastric cancer. *Br J Surg* **84**: 133–136

Yu AE, Hewitt RE, Connor EW and Stetler Stevenson WG (1997) Matrix metalloproteinases: novel targets for directed cancer therapy. *Drugs Ageing* **11**: 229–244

© 1999 Cancer Research Campaign
